# Sexual Dysfunction in Female Medical Residents: The Influence of Dispositional Mindfulness

**DOI:** 10.1192/j.eurpsy.2025.2286

**Published:** 2025-08-26

**Authors:** R. Masmoudi, S. Ajmi, M. Mnif, F. Guermazi, I. Feki, I. Baati, J. Masmoudi

**Affiliations:** 1 Psychiatry A department, Hedi Chaker hospital university, Sfax, Tunisia

## Abstract

**Introduction:**

Dispositional mindfulness (DM), also known as Trait mindfulness, refers to the inherent ability to focus on and sustain attention on present-moment experiences with an open and nonjudgmental attitude. Recent research has increasingly highlighted the positive impact of DM on mental health.

**Objectives:**

To evaluate the association between DM and sexual dysfunction (SD) among female medical residents.

**Methods:**

A cross-sectional online survey, designed using Google Forms, was distributed on social media platforms. We included married and sexually active medical residents. Female Sexual Function Index (FSFI) was used to evaluate female sexual dysfunction. We used the Mindfulness Attention Awareness Scale (M.A.A.S.) to measure the frequency of open and receptive attention and awareness of ongoing events and experiences and the Spielberger State-Trait Anxiety Inventory (STAI-Y-T) to assess anxiety traits.

**Results:**

Sixty-five medical residents with an average age of 29.43 ± 1.95 years completed the online questionnaire. The average age at first sexual intercourse was 25.15 ± 2.34 years and the average frequency of sexual intercourse was 6.61 ± 4.18 times per month.

The mean scores for the M.A.A.S and STAI-Y-T were 26.25 ±5.61 and 45.85 ±9.36 respectively.

The anxiety assessment using the STAI-Y-T scale showed that 30 residents (46.4%) had moderate anxiety, and 8 (12.3%) had high to very high anxiety levels.

The mean total FSFI score was 26.25 ± 5.61 and SD was noted in 49.2% of the cases.

A positive correlation was found between the level of MAAS and the total FSFI score (p=0.003; r=0.35),”Desire” dimension (p=0.02; r=0.28), “Lubrication” domain (p=0.004; r=0.35), and the “Satisfaction” (p=0.006; r=0.33)

Anxiety levels were negatively correlated with the total FSFI score (p<10^-3^; r=-0.42), the “Desire” dimension (p=0.008; r=-0.32), “Arousal” dimension (p=0.003; r=-0.36), “Lubrication” domain (p=0.002; r=-0.37) and “Satisfaction” domain (p=0.002; r=-0.38).

**Conclusions:**

The results of this study revealed relatively high prevalence rates of SD among medical residents and highlighted several vulnerability factors. Therefore, several measures should be implemented to prevent and determine the factors correlated with SD.

**Disclosure of Interest:**

None Declared

